# Differences among patients with and without nonalcoholic fatty liver disease having elevated alanine aminotransferase levels at various stages of metabolic syndrome

**DOI:** 10.1371/journal.pone.0238388

**Published:** 2020-08-31

**Authors:** Masahiro Sogabe, Toshiya Okahisa, Takeshi Kurihara, Masanori Takehara, Kaizo Kagemoto, Jun Okazaki, Yoshifumi Kida, Akihiro Hirao, Hironori Tanaka, Tetsu Tomonari, Tatsuya Taniguchi, Koichi Okamoto, Masahiko Nakasono, Tetsuji Takayama

**Affiliations:** 1 Department of Gastroenterology and Oncology, Tokushima University Graduate School of Biomedical Sciences, Tokushima, Japan; 2 Department of Internal Medicine, Shikoku Central Hospital of the Mutual aid Association of Public School Teachers, Shikokuchuo, Japan; 3 Department of Internal Medicine, Tsurugi Municipal Handa Hospital, Tsurugi, Japan; East Tennessee State University, UNITED STATES

## Abstract

**Background:**

The prevalence of nonalcoholic fatty liver disease (NAFLD) in the non-obese population has increased and NAFLD is not always recognized in individuals with metabolic syndrome (MS). The risk of cirrhosis is higher in patients having NAFLD with elevated alanine aminotransferase (ALT) levels than in those having NAFLD with normal ALT levels.

**Objective:**

To measure the differences in clinical factors associated with NAFLD having elevation of ALT among subjects with Non-MS, Pre-MS, and MS, and to measure differences in metabolites between MS subjects with and without NAFLD having elevation of ALT.

**Methods:**

Among 7,054 persons undergoing health check-ups, we included 3,025 subjects who met the selection criteria. We measured differences in clinical factors for NAFLD having elevation of ALT among subjects with Non-MS, Pre-MS, and MS, and compared metabolites between subjects with and without NAFLD having elevation of ALT in 32 subjects with MS.

**Results:**

The prevalence of NAFLD and NAFLD having elevation of ALT was significantly progressively greater in subjects with Non-MS, Pre-MS, and MS (*p* <0.001, respectively). In the Non-MS group, there were significant differences between subjects with and without NAFLD having elevation of ALT with respect to body mass index (BMI), high-density lipoprotein cholesterol (HDL-C), low-density lipoprotein cholesterol, hemoglobin A1c, uric acid, aspartate aminotransferase (AST); In the Pre-MS group, there were significant differences in BMI, hypertension, AST, and gamma-glutamyl transpeptidase (GGT); In the MS group, there were significant differences in HDL-C, impaired glucose tolerance, AST, and GGT. There were significant differences in levels of metabolites of nicotinamide, inosine, and acetyl-L-carnitine between MS subjects with and without NAFLD having elevation of ALT (all *p* <0.05).

**Conclusions:**

Although NAFLD having elevation of ALT is important for development of NAFLD, differences in factors associated with NAFLD having elevation of ALT at various stages of MS should be considered. Additionally, several metabolites may play roles in the identification of risk for NAFLD in individuals with MS.

## Introduction

Despite the fact that the increase in prevalence of metabolic syndrome (MS) that is strongly associated with nonalcoholic fatty liver disease (NAFLD) has been problematic in gastroenterology, NAFLD is not always recognized in individuals with MS [1–3]. Persons who are easy to become NAFLD and those who are hard to become NAFLD may exist in individuals who belong to the same MS. Additionally, the prevalence of NAFLD in the non-obese population has gradually increased in Japan, and not low [4, 5]. Generally, NAFLD is diagnosed using the presence of fatty liver regardless of assessment of liver enzymes in the context of medical check-ups and NAFLD having elevation of liver enzyme and NAFLD having standard values of liver enzyme have been treated in the same way; nevertheless, patients with NAFLD and elevation of alanine aminotransferase (ALT) are at higher risk for cirrhosis than those with NAFLD and normal ALT values [6]. However, there is few reports about NAFLD having elevation of ALT levels at various stages of MS. The aim of this study was to measure the differences in clinical factors associated with NAFLD having elevation of ALT among subjects with Non-MS, Pre-MS, and MS, and to measure differences in metabolites between MS subjects with and without NAFLD having elevation of ALT.

## Methods

### Study design and subjects

**Fig 1:**
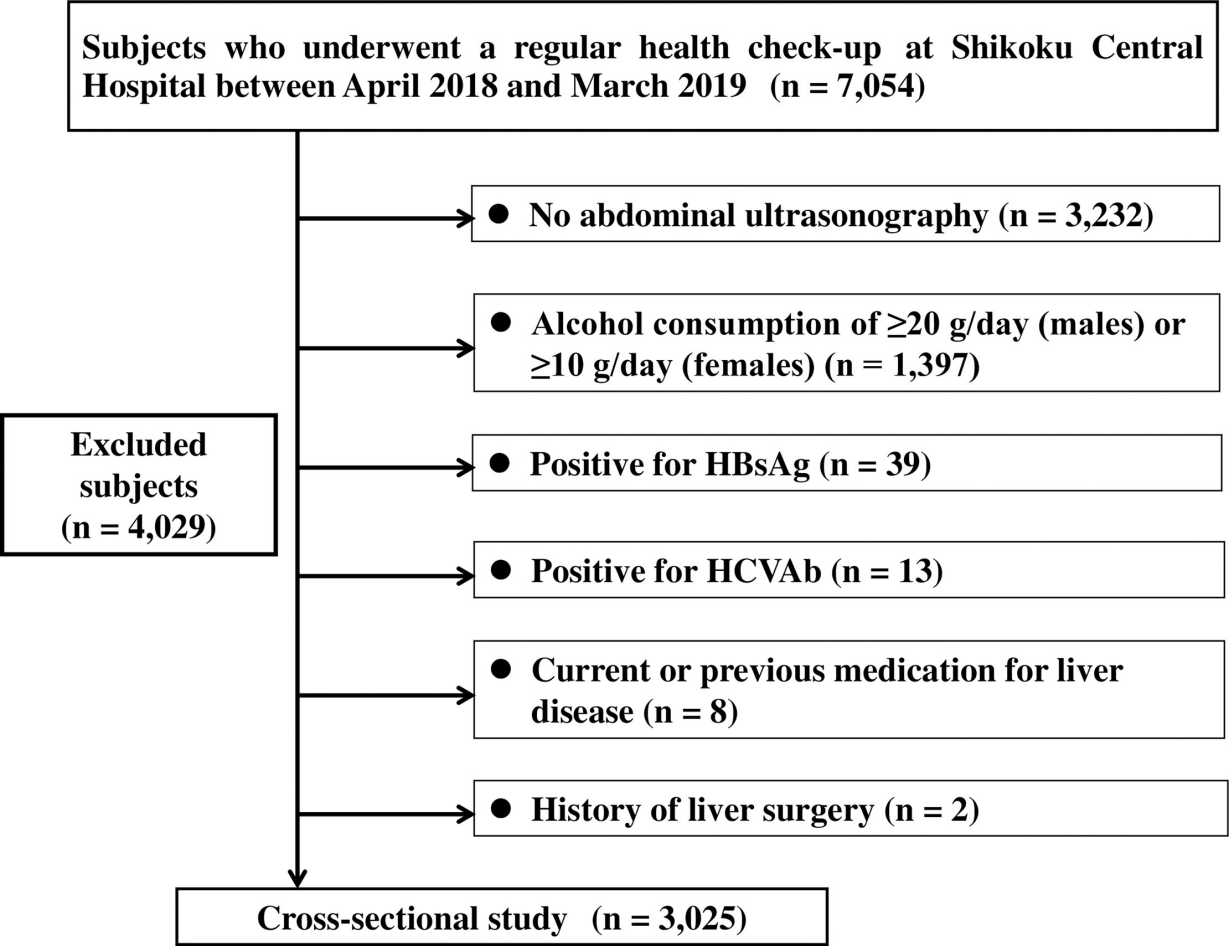
Participant flow of individuals undergoing check-ups. HBsAg, hepatitis B surface antigen; HCVAb, hepatitis C antibody.

This cross-sectional study was conducted among 7,054 subjects residing in the Shikoku region, Japan, and undergoing regular health check-ups at Shikoku Central Hospital of the Mutual Aid Association of Public School Teachers between April 2018 and March 2019. Among 7,054 subjects, 4,029 subjects were excluded for fulfilling any of the following criteria: (1) positivity for markers of hepatitis B virus infection (hepatitis B surface antigen) and/or hepatitis C virus infection (anti-hepatitis C virus antibodies); (2) alcohol consumption of ≥20 g/day in males or alcohol consumption of ≥10 g/day in females; (3) absence of abdominal ultrasonography; (4) history of liver surgery; and (5) current or previous medication for liver disease. Finally, 3,025 subjects were enrolled (Fig 1). The study protocol was approved by the Ethics Committee of Shikoku Central Hospital, and all procedures were performed in accordance with the Declaration of Helsinki. All subjects were informed that their clinical data might be retrospectively analyzed, and informed consent was obtained.

### Diagnosis of MS

The diagnostic criteria for MS adopted by the World Health Organization (WHO) or the adult treatment panel (ATP) III criteria are used worldwide. However, we adopted the MS criteria proposed by the joint committee of eight Japanese medical societies in 2005 because all subjects in our study were Japanese [7]. Component factors of MS criteria are waist circumference (WC) must exceed 85 cm for males or 90 cm for females, and the presence of two or more of the following: (1) dyslipidemia: high-density lipoprotein cholesterol (HDL-C) *<*40 mg/dl, and/or triglycerides (TG) ≥150 mg/dl, or medication for dyslipidemia; (2) impaired glucose tolerance (IGT): fasting plasma glucose (FPG) ≥110 mg/dl or medication for diabetes; and (3) hypertension: blood pressure ≥ 130/85 mmHg or medication for hypertension.

We designated individuals who fulfilled these criteria as the MS group. Individuals who did not fulfill above MS criteria were divided into two groups as follows: The Non-MS group was defined as individuals having no component of MS; The Pre-MS group was defined as individuals having more than 85 cm of WC for males or 90 cm for females and one component of MS.

### Physical examination and serum biochemistry

Body weight (BW) and height were obtained from all subjects. BW was measured to the nearest 0.1 kg, and height was measured to the nearest 0.1 cm. The body mass index (BMI) was calculated as the weight (in kilograms) divided by the square of the height (in meters) expressed in kg/m^2^. WC was measured at the umbilical level by a laboratory technician. Venous blood samples were taken from all subjects in the morning after 12 hours of overnight fasting. Clinical laboratory tests included aspartate aminotransferase (AST), ALT, gamma-glutamyl transpeptidase (GGT), total cholesterol (T-CHO), HDL-C, TG, low-density lipoprotein cholesterol (LDL-C), uric acid (UA), FPG, and hemoglobin A1c (HbA1c). Ferritin, insulin, and type IV collagen 7S were measured among 34 subjects with MS during 2018 April, and the homeostasis model assessment of insulin resistance (HOMA-IR) and NAFIC score and the Fibrosis (FIB)-4 indexes were assessed [8–12]. ALT elevation was defined as level more than 30 IU/L.

### Assessment of ultrasonography

Standard abdominal ultrasonography was performed by trained technicians in the morning with the subjects fasting. The HI-VISION Avius^®^ and ALOKA ARIETTA 850 (Hitachi Ltd., Tokyo, Japan) platforms with 6-MHz convex-array probes were used for ultrasonography. The diagnostic criteria for fatty liver on ultrasonography were as follows: echo contrast between the liver and the renal cortex, liver brightness, blurring of liver vessels, and/or deep attenuation [13, 14]. In our study, fatty liver with alcohol consumption of less than 20 g/day in males or alcohol consumption of less than 10 g/day in females and without chronic liver diseases such as hepatitis B, hepatitis C, and liver disease related with autoimmune was defined as NAFLD.

### Metabolomics and sulfur metabolomics

Sera from 34 subjects with MS during 2018 April were obtained by centrifugation of blood samples for 10 min at 1500× *g* at 4°C, and was stored at –80°C until use. Metabolomics following the primary metabolism method was performed using liquid chromatography coupled to a tandem mass spectrometry (LC-MS/MS) (Nexera UHPLC system with on-line LC-MS 8040, Shimadzu Corporation, Kyoto, Japan). Briefly, 10 μL of serum was added to 110 μL methanol containing internal standards at 0°C to inactivate native enzymes. After centrifugation, the upper aqueous layer of the separated solution was desiccated using a centrifugal evaporator. Thereafter, 60 μL of water was added, and the solution was centrifuged. We used 10 μL from the upper aqueous layer as the metabolomics sample. Sulfur metabolomics was performed by the Sulfur Index service in Japan (http://www.euglena.jp/sulfurindex/; Euglena Co. Ltd., Tokyo, Japan). The sulfur index uses a technology where metabolites are detected as S-bimanyl derivatives by LC-MS/MS (Shimadzu (Nexera UHPLC system with on-line LC-MS 8040, Shimadzu, Corporation, Kyoto, Japan) as described previously [15, 16]. Briefly, the sulfur-containing compounds in the samples were extracted by adding methanol and converting to fluorescent derivatives with monobromobimane. The levels of the target metabolites were determined from the peak area in the mass chromatography, monitoring each mass-to-charge ratio of the individual target, and represented as relative amounts (relative areas) after normalization based on the peak area of the internal standard (D-camphor-10-sulfonic acid). We measured 101 primary metabolites such as amino acids, organic acids, and the like, and a total of 52 metabolites were obtained from subjects with MS. We measured 90 sulfur metabolites and found a total of 17 sulfur metabolites in subjects with MS.

### Statistical analysis

Quantitative data, including subject baseline characteristics, were expressed as the mean ± standard deviation (SD). *P-*values of less than 0.05 were considered statistically significant. The χ^2^-test or Mann–Whitney U-test was used for comparisons between the two groups. The m × n χ^2^-test or Kruskal–Wallis test was used to analyze differences among three groups. If the Kruskal–Wallis test revealed differences between the groups, then post-hoc pairwise comparisons were performed using the Mann–Whitney U test with Bonferroni correction. Correlations between variables were assessed by calculating Spearman rank correlation coefficients. Factors with significant influence on the prevalence of NAFLD having elevation of ALT were determined using univariate analysis. All parameters that had *P-*values of less than 0.05 by univariate analysis were assessed using stepwise multivariate logistic regression analysis adjusted for age and gender. The odds ratio (OR) and 95% confidence interval (CI) were analyzed for each variable. All statistical analyses were performed using MedCalc Statistical Software for Windows (MedCalc Software; Ostend, Belgium).

## Results

### Baseline characteristics among Non-MS, Pre-MS, and MS groups

The baseline characteristics of 3,025 subjects are shown in Table 1. The prevalence of Non-MS, Pre-MS, and MS groups was 74.3%, 11.9%, and 13.8%, respectively. The proportion of males, age, BMI, and WC were significantly high in order of MS group, Pre-MS group, Non-MS group (*p* < 0.001, *p* <0.05, *p* <0.05, and *p* <0.05, respectively). The prevalence of smoker and drinker in MS group and Pre-MS group were significantly higher than in Non-MS group (*p* < 0.001 and *p* <0.05, respectively). The prevalence of hypertension, SBP, and DBP were significantly high in order of MS group, Pre-MS group, Non-MS group (*p* < 0.001, *p* <0.05, and *p* <0.05, respectively). The prevalence of dyslipidemia, TG, and LDL-C were significantly high in order of MS group, Pre-MS group, Non-MS group (*p* < 0.001, *p* <0.05, and *p* <0.05, respectively), and HDL-C was significantly low in order of MS group, Pre-MS group, Non-MS group (*p* <0.05). FPG and HbA1c were significantly high in order of MS group, Pre-MS group, Non-MS group (*p* <0.05 for both). The prevalence of IGT was significantly higher in MS group than in Pre-MS group and Non-MS group (*p* <0.001). UA was significantly high in order of MS group, Pre-MS group, Non-MS group (*p* <0.05). ALT, AST, GGT, and the prevalence of NAFLD were significantly high in order of MS group, Pre-MS group, Non-MS group (*p* < 0.05, *p* <0.05, *p* <0.05, and *p* <0.001, respectively).

**Table 1:** Baseline characteristics among Non-MS, Pre-MS, and MS groups (n = 3,025).

	Total	Non-MS	Pre-MS	MS	*p*-value
	subjects	group	group	group	
	(n = 3,025)	(n = 2,246)	(n = 361)	(n = 418)	
Gender (F/M)	1,739/1,286	1506/740	125/236	108/310	< 0.001°(*<0.001, ^§^<0.001, °<0.001)
Age (years)	52.0 ± 9.4	51.2 ± 9.7^a^	53.5 ± 8.1^b^	55.4 ± 7.2^c^	< 0.001
BMI (kg/m^2^)	23.6 ± 3.9	22.1 ± 2.8^a^	27.2 ± 3.4^b^	28.2 ± 3.8^c^	< 0.001
WC (cm)	83.2 ± 10.4	79.2 ± 7.7^a^	93.7 ± 7.2^b^	96.1 ± 8.5^c^	< 0.001
Smoker, n (%)	270 (8.9%)	155 (6.9%)	52 (14.4%)	63 (15.1%)	< 0.001 (*<0.001, °<0.001)
Drinker, n (%)	1,278 (42.2%)	906 (40.3%)	176 (48.8%)	196 (46.9%)	< 0.005 (*<0.05, °<0.005)
SBP (mmHg)	124 ± 17	120 ± 16^a^	132 ± 16^b^	137 ± 16^c^	< 0.001
DBP (mmHg)	77 ± 13	74 ± 11^a^	83 ± 11^b^	88 ± 12^c^	< 0.001
Hypertension, n (%)	1,276 (42.2%)	649 (28.9%)	240 (66.5%)	387 (92.6%)	< 0.001 (*<0.001, ^§^<0.001, °<0.001)
T-CHO (mg/dL)	210.8 ± 35.0	210.7 ± 34.8	213.1 ± 33.2	209.1 ± 37.3	0.253
TG (mg/dL)	104.7 ± 69.3	89.7 ± 51.1^a^	121.5 ± 56.6^b^	170.7 ± 110.2^c^	< 0.001
HDL-C (mg/dL)	66.5 ± 17.4	70.5 ± 17.0^a^	57.2 ± 12.3^b^	53.0 ± 13.3^c^	< 0.001
LDL-C (mg/dL)	128.7 ± 30.6	126.9 ± 30.1^a^	137.0 ± 29.4^b^	131.2 ± 32.6^c^	< 0.001
Dyslipidemia, n (%)	797 (26.3%)	341 (15.2%)	103 (28.5%)	353 (84.4%)	< 0.001 (*<0.001, ^§^<0.001, °<0.001)
FPG (mg/dL)	100.3 ± 16.8	96.7 ± 11.2^a^	100.9 ± 9.7^b^	118.7 ± 29.7^c^	< 0.001
HbA1c (%)	5.6 ± 0.6	5.5 ± 0.4^a^	5.6 ± 0.4^b^	6.2 ± 1.0^c^	< 0.001
IGT, n (%)	445 (14.7%)	193 (8.6%)	18 (5.0%)	234 (56.0%)	< 0.001 (*<0.001, ^§^<0.001)
UA (mg/dL)	5.0 ± 1.3	4.8 ± 1.2^a^	5.8 ± 1.3^b^	5.9 ± 1.3^c^	< 0.001
ALT (IU/L)	23.0 ± 16.3	19.3 ± 11.4^a^	29.5 ± 17.7^b^	37.0 ± 25.3^c^	< 0.001
AST (IU/L)	23.7 ± 8.9	22.3 ± 6.6^a^	25.4 ± 8.8^b^	29.6 ± 15.1^c^	< 0.001
GGT (IU/L)	30.4 ± 29.9	26.2 ± 27.0^a^	36.6 ± 27.9^b^	48.0 ± 37.5^c^	< 0.001
NAFLD, n (%)	1,002 (33.1%)	417 (18.6%)	242 (67.0%)	343 (82.1%)	< 0.001 (*<0.001, ^§^<0.001, °<0.001)

Data represent the mean ± standard deviation (SD) and number for categorical variables.

*P*-values are based on the m × n χ^2^-test or Kruskal Wallis test. If the Kruskal Wallis test revealed differences between the groups, then post-hoc pairwise comparisons were performed using the Mann-Whitney U test with Bonferroni correction. Different letters (a, b, c) indicate a significant difference at the 0.0166 (0.05/3) level. The χ^2^-test was used for comparisons of number for categorical variables between the two groups (*MS group vs. non-MS group, ^§^MS group vs. pre-MS group, °pre-MS group vs. non-MS group). Significant is at the 5% level.

ALT, alanine aminotransferase; AST, aspartate aminotransferase; BMI, body mass index; DBP, diastolic blood pressure; F, female; FPG, fasting plasma glucose; GGT, gamma-glutamyl transpeptidase; HbA1c, hemoglobin A1c; HDL-C, high-density lipoprotein cholesterol; IGT, impaired glucose tolerance; LDL-C, low-density lipoprotein cholesterol; M, male; MS, metabolic syndrome; NAFLD, nonalcoholic fatty liver disease; SBP, systolic blood pressure; T-CHO, total cholesterol; TG, triglyceride; UA, uric acid; WC, waist circumference. Drinker: < 20 g/day of alcohol in male; < 10 g/day of alcohol in female.

### Comparison of the prevalence of NAFLD and NAFLD having elevation of ALT among Non-MS, Pre-MS, and MS groups

**Fig 2:**
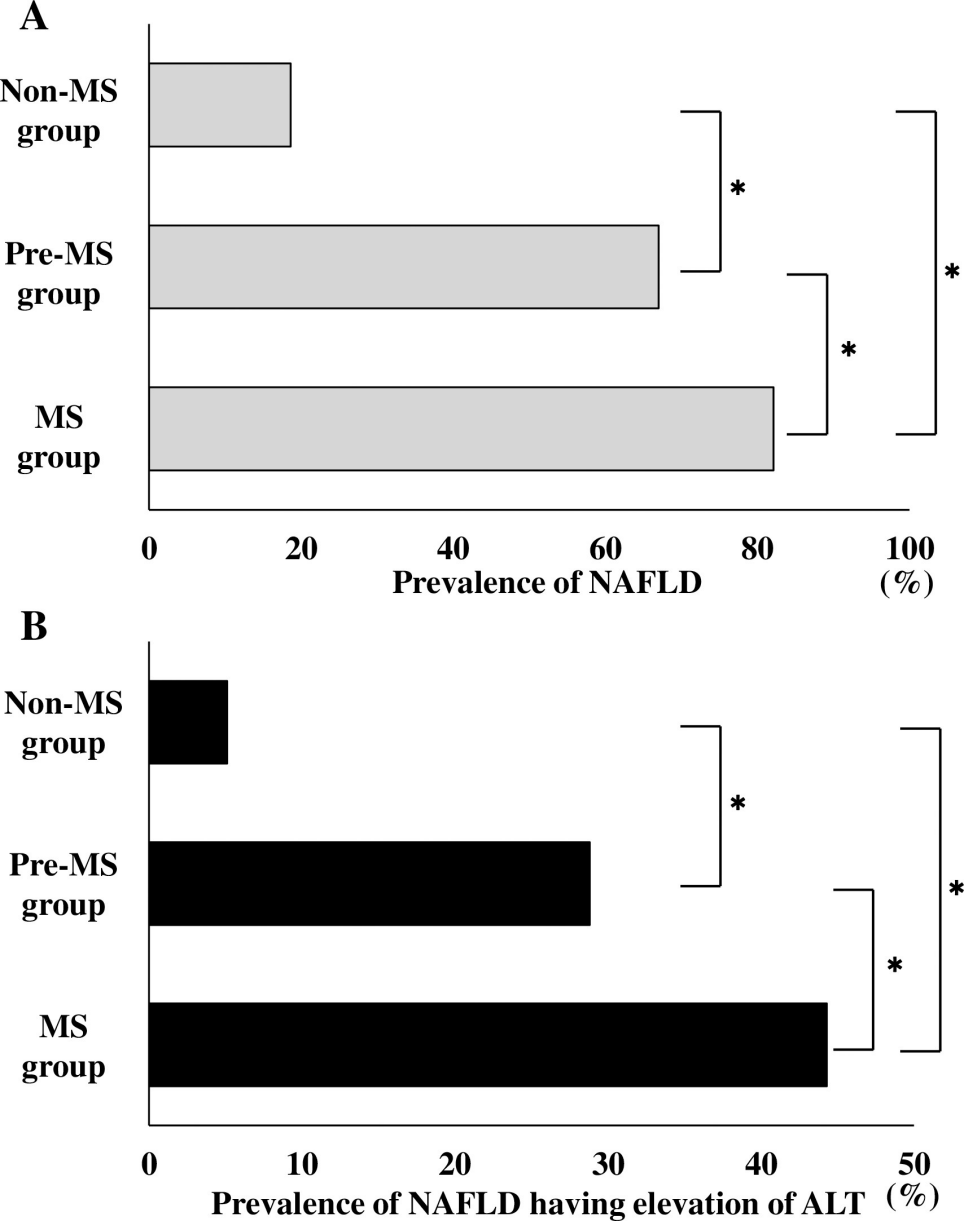
Comparison of the prevalence of NAFLD and NAFLD having elevation of ALT among the Non-MS, Pre-MS, and MS groups. **A** Comparison of the prevalence of NAFLD among the Non-MS, Pre-MS, and MS groups. **B** Comparison of the prevalence of NAFLD having elevation of ALT among the Non-MS, Pre-MS, and MS groups. The gray bar indicates the prevalence of NAFLD. The black bar indicates the prevalence of NAFLD having elevation of ALT. ALT, alanine aminotransferase; MS, metabolic syndrome; NAFLD, nonalcoholic fatty liver disease; *P < 0.001.

The comparison of the prevalence of NAFLD among the Non-MS, Pre-MS, and MS groups is shown in Fig 2A. The prevalence of NAFLD in Non-MS group, Pre-MS group, and MS group were 18.6% (417/2,246), 67.0% (242/361), and 82.1% (343/418), respectively. There was a significant difference in the prevalence of NAFLD among the three groups (*p* < 0.001). The prevalence of NAFLD was higher in the MS group vs. the Pre-MS group and the Non-MS group (*p* < 0.001 and *p* < 0.001, respectively), and the prevalence of NAFLD was significantly higher in the Pre-MS group than in the Non-MS group (*p* <0.001).

A comparison of the prevalence of NAFLD having elevation of ALT among Non-MS, Pre-MS, and MS groups is shown in Fig 2B. The prevalence of NAFLD having elevation of ALT in Non-MS, Pre-MS, and MS groups were 5.1% (115/2,246), 28.8% (104/361), and 44.3% (185/418), respectively. There were significant differences in the prevalence of NAFLD having elevation of ALT among the three groups (*p* < 0.001). The prevalence of NAFLD having elevation of ALT was higher in the MS group than in the Pre-MS group and Non-MS group (*p* < 0.001 and *p* < 0.001, respectively), and the prevalence of NAFLD having elevation of ALT was higher in Pre-MS group than in the Non-MS group (*p* <0.001).

### Factors associated with NAFLD having elevation of ALT among Non-MS, Pre-MS, and MS groups

Results of univariate analysis for factors associated with NAFLD having elevation of ALT among Non-MS group, Pre-MS group, and MS group are shown in Table 2. In the Non-MS group, univariate analysis showed that gender, BMI, WC, SBP, DBP, hypertension, T-CHO, TG, HDL-C, LDL-C, dyslipidemia, FPG, HbA1c, IGT, UA, AST, and GGT were significantly associated with NAFLD having elevation of ALT. In the Pre-MS group, univariate analysis showed that gender, age, BMI, WC, hypertension, HDL-C, dyslipidemia, FPG, HbA1c, UA, AST, and GGT were significantly associated with NAFLD having elevation of ALT. In the MS group, univariate analysis showed that gender, age, BMI, WC, TG, HDL-C, HbA1c, IGT, UA, AST, and GGT were significantly associated with NAFLD having elevation of ALT.

**Table 2:** Results of univariate analysis for factors associated with NAFLD having elevation of ALT among Non-MS, Pre-MS, and MS groups.

		Non-MS group		Pre-MS group		MS group	
Factor		OR (95% CI)	*p*-value	OR (95% CI)	*p*-value	OR (95% CI)	*p*-value
Gender		4.880 (3.256–7.315)	< 0.001	2.901 (1.678–5.016)	< 0.001	1.753 (1.112–2.765)	< 0.05
Age	(years)	1.000 (0.981–1.020)	0.999	0.950 (0.923–0.978)	< 0.001	0.954 (0.927–0.981)	< 0.005
BMI	(kg/m^2^)	1.417 (1.329–1.511)	< 0.001	1.123 (1.050–1.200)	< 0.001	1.165 (1.099–1.235)	< 0.001
WC	(cm)	1.139 (1.110–1.169)	< 0.001	1.046 (1.014–1.079)	< 0.005	1.067 (1.040–1.095)	< 0.001
SBP	(mmHg)	1.019 (1.008–1.031)	< 0.001	0.995 (0.981–1.010)	0.498	0.998 (0.986–1.010)	0.715
DBP	(mmHg)	1.036 (1.020–1.052)	< 0.001	1.000 (0.980–1.021)	0.999	1.013 (0.997–1.030)	0.123
Hypertension		1.824 (1.244–2.674)	< 0.005	0.551 (0.344–0.882)	< 0.05	1.108 (0.528–2.324)	0.787
T-CHO	(mg/dL)	1.007 (1.002–1.012)	< 0.01	0.998 (0.991–1.005)	0.610	1.001 (0.995–1.006)	0.836
TG	(mg/dL)	1.011 (1.008–1.014)	< 0.001	1.002 (0.998–1.006)	0.310	1.003 (1.001–1.005)	< 0.005
HDL-C	(mg/dL)	0.930 (0.915–0.944)	< 0.001	0.961 (0.941–0.982)	< 0.001	0.966 (0.950–0.983)	< 0.001
LDL-C	(mg/dL)	1.016 (1.010–1.022)	< 0.001	1.002 (0.995–1.010)	0.568	1.001 (0.995–1.007)	0.704
Dyslipidemia		3.382 (2.265–5.048)	< 0.001	1.701 (1.044–2.771)	< 0.05	0.913 (0.537–1.553)	0.738
FPG	(mg/dL)	1.041 (1.028–1.054)	< 0.001	1.024 (1.000–1.048)	< 0.05	1.005 (0.998–1.012)	0.142
HbA1c	(%)	3.376 (2.346–4.860)	< 0.001	1.851 (1.064–3.221)	< 0.05	1.296 (1.060–1.585)	< 0.05
IGT		3.062 (1.901–4.931)	< 0.001	1.614 (0.608–4.284)	0.337	1.574 (1.063–2.331)	< 0.05
UA	(mg/dL)	2.025 (1.748–2.346)	< 0.001	1.240 (1.031–1.490)	< 0.05	1.315 (1.125–1.536)	< 0.001
AST	(IU/L)	1.206 (1.173–1.240)	< 0.001	1.283 (1.212–1.358)	< 0.001	1.243 (1.187–1.301)	< 0.001
GGT	(IU/L)	1.020 (1.016–1.025)	< 0.001	1.030 (1.018–1.041)	< 0.001	1.040 (1.029–1.051)	< 0.001
FIB-4 index		0.748 (0.491–1.140)	0.173	0.652 (0.382–1.112)	0.652	1.338 (0.883–2.029)	0.170
Smoker		1.620 (0.870–3.015)	0.150	1.239 (0.660–2.328)	0.505	0.802 (0.464–1.385)	0.428
Drinker		0.878 (0.596–1.293)	0.509	1.195 (0.758–1.886)	0.444	0.864 (0.587–1.273)	0.460

ALT, alanine aminotransferase; AST, aspartate aminotransferase; BMI, body mass index; CI, confidence interval; DBP, diastolic blood pressure; FPG, fasting plasma glucose; GGT, gamma-glutamyl transpeptidase; HbA1c, hemoglobin A1c; HDL-C, high-density lipoprotein cholesterol; IGT, impaired glucose tolerance; LDL-C, low-density lipoprotein cholesterol; MS, metabolic syndrome; NAFLD, nonalcoholic fatty liver disease; OR, odds ratio; SBP, systolic blood pressure; T-CHO, total cholesterol; TG, triglyceride; UA, uric acid; WC, waist circumference. Drinker: < 20 g/day of alcohol in male; < 10 g/day of alcohol in female.

### Independent predictors of NAFLD having elevation of ALT among Non-MS, Pre-MS, and MS groups

Results of multivariate analysis for independent predictors of NAFLD having elevation of ALT among Non-MS group, Pre-MS group, and MS group are shown in Table 3. In the Non-MS group, BMI, LDL-C, HbA1c, UA and AST were significant independent predictors of increased prevalence of NAFLD having elevation of ALT, whereas HDL-C contributed significantly and independently to decreased prevalence of NAFLD having elevation of ALT. In the Pre-MS group, BMI, AST, and GGT were significant and independent predictors of increased prevalence of NAFLD having elevation of ALT, whereas hypertension contributed significantly and independently to decreased prevalence of NAFLD having elevation of ALT. In the MS group, IGT, AST, and GGT were significant and independent predictors of increased prevalence of NAFLD having elevation of ALT, whereas HDL-C contributed significantly and independently to decreased prevalence of NAFLD having elevation of ALT.

**Table 3:** Results of multivariate analysis for independent predictors of NAFLD having elevation of ALT among Non-MS, Pre-MS, and MS groups.

	Non-MS group		Pre-MS group		MS group	
Factor	OR (95% CI)	*p-*value	OR (95% CI)	*p-*value	OR (95% CI)	*p-*value
BMI (kg/m^2^)	1.358 (1.232–1.496)	< 0.001	1.179 (1.059–1.312)	< 0.005		
Hypertension			0.416 (0.937–1.004)	< 0.05		
HDL-C (mg/dL)	0.955 (0.934–0.975)	< 0.001			0.963 (0.941–0.987)	< 0.005
LDL-C (mg/dL)	1.012 (1.005–1.020)	< 0.005				
HbA1c (%)	2.473 (1.531–3.995)	< 0.001				
IGT					2.605 (1.469–4.621)	< 0.005
UA (mg/dL)	1.315 (1.061–1.631)	< 0.05				
AST (IU/L)	1.211 (1.171–1.252)	< 0.001	1.310 (1.224–1.402)	< 0.001	1.240 (1.178–1.305)	< 0.001
GGT (IU/L)			1.018 (1.003–1.033)	< 0.05	1.021 (1.021–1.033)	< 0.001

All the parameters that had a *P* value of less than 0.05 by univariate analysis were assessed using stepwise multivariate logistic regression analysis adjusted age and gender.

ALT, alanine aminotransferase; AST, aspartate aminotransferase; BMI, body mass index; CI, confidence interval; GGT, gamma-glutamyl transpeptidase; HbA1c, hemoglobin A1c; HDL-C, high-density lipoprotein cholesterol; IGT, impaired glucose tolerance; LDL-C, low-density lipoprotein cholesterol; MS, metabolic syndrome; NAFLD, nonalcoholic fatty liver disease; OR, odds ratio; UA, uric acid.

### Comparison of baseline characteristics between subjects with and without NAFLD having elevation of ALT in 32 subjects with MS

A comparison of the baseline characteristics between subjects with and without NAFLD having elevation of ALT in 32 subjects with MS are shown in Table 4. The mean age in subjects without NAFLD having elevation of ALT was significantly higher than that in subjects with NAFLD having elevation of ALT (*p* < 0.05). AST, HOMA-IR, and NAFIC scores in subjects with NAFLD having elevation of ALT were significantly higher than those in subjects without NAFLD having elevation of ALT (*p* < 0.001, *p* < 0.05, and *p* < 0.005, respectively).

**Table 4:** Comparison of baseline characteristics between subjects with and without NAFLD having elevation of ALT in 32 subjects with MS.

	Subjects without NAFLD having elevation of ALT (n = 14)	Subjects with NAFLD having elevation of ALT (n = 18)	*p*-value
Gender (F/M) (%M)	3/11 (78.6%)	6/12 (66.7%)	0.729
Age (years)	57.4 ± 3.1	54.3 ± 4.2	< 0.05
BMI (kg/m^2^)	27.9 ± 5.0	29.0 ± 5.6	0.372
WC (cm)	95.9 ± 9.9	98.0 ± 11.9	0.447
Smoker, n (%)	2 (14.3%)	4 (22.2%)	0.909
Drinker, n (%)	8 (57.1%)	9 (50.0%)	0.964
SBP (mmHg)	142 ± 24	143 ± 20	0.894
DBP (mmHg)	91 ± 11	97 ± 11	0.098
Hypertension, n (%)	12 (78.6%)	16 (88.9%)	0.788
T-CHO (mg/dL)	210.4 ± 43.4	216.8 ± 45.2	0.447
TG (mg/dL)	154.9 ± 95.3	159.3 ± 124.3	0.849
HDL-C (mg/dL)	61.9 ± 13.0	57.6 ± 11.7	0.333
LDL-C (mg/dL)	128.9 ± 37.6	136.1 ± 45.9	0.531
Dyslipidemia, n (%)	11 (78.6%)	9 (50.0%)	0.198
FPG (mg/dL)	113.4 ± 35.0	107.1 ± 13.5	0.733
HbA1c (%)	6.2 ± 0.9	5.9 ± 0.4	0.608
IGT, n (%)	5 (35.7%)	6 (33.3%)	0.815
UA (mg/dL)	6.2 ± 1.1	5.8 ± 1.9	0.323
AST (IU/L)	23.2 ± 6.3	37.1 ± 11.3	< 0.001
GGT (IU/L)	73.8 ± 136.3	117.9 ± 109.9	< 0.05
HOMA-IR	1.37 ± 0.68	2.14 ± 1.22	< 0.05
NAFIC score	0.00 ± 0.00	0.78 ± 0.94	< 0.005
FIB-4 index	1.24 ± 0.46	1.29 ± 0.43	0.536

Data represent the mean ± standard deviation (SD) and number for categorical variables. All *p*-values are based on the χ^2^-test or Mann-Whitney U-test. Significant is at the 5% level.

ALT, alanine aminotransferase; AST, aspartate aminotransferase; BMI, body mass index; DBP, diastolic blood pressure; F, female; FPG, fasting plasma glucose; GGT, gamma-glutamyl transpeptidase; HbA1c, hemoglobin A1c; HDL-C, high-density lipoprotein cholesterol; HOMA-IR, homeostasis model assessment of insulin resistance; IGT, impaired glucose tolerance; LDL-C, low-density lipoprotein cholesterol; M, male; MS, metabolic syndrome; NAFLD, nonalcoholic fatty liver disease; SBP, systolic blood pressure; T-CHO, total cholesterol; TG, triglyceride; UA, uric acid; WC, waist circumference. Drinkers: < 20 g/day of alcohol in male; < 10 g/day of alcohol in female.

### Comparison of primary metabolites between subjects with and without NAFLD having elevation of ALT in 32 subjects with MS

A comparison of 52 metabolites between subjects with and without NAFLD having elevation of ALT in 32 subjects with MS are shown in Table 5. There were significant differences in three of the 52 metabolites between subjects with and without NAFLD having elevation of ALT. Mean nicotinamide levels in the subjects without NAFLD having elevation of ALT and subjects with NAFLD having elevation of ALT of ALT were 2.860E-04 ± 1.064E-04 and 3.877E-04 ± 1.284E-04, respectively, which was a statistically significant difference (*p* < 0.05). Mean inosine levels in the subjects without NAFLD having elevation of ALT and subjects with NAFLD having elevation of ALT were 0 and 2.646E-05 ± 4.495E-05, respectively, which was also a statistically significant difference (*p* < 0.05). Mean acetyl-L-carnitine levels in the subjects without NAFLD having elevation of ALT and subjects with NAFLD having elevation of ALT were 1.013E-01 ± 1.947E-02 and 8.699E-02 ± 1.874E-02, respectively, which was also a statistically significant difference (*p* < 0.05).

**Table 5:** Comparison of 52 metabolites between subjects with and without NAFLD having elevation of ALT in 32 subjects with MS.

Compound name	Relative area				
	Subjects without NAFLD having elevation of ALT (n = 14)		Subjects with NAFLD having elevation of ALT (n = 18)		*p*-value
	Mean	SD	Mean	SD	
Cystine	1.082E-03	2.017E-04	1.028E-03	1.389E-04	0.640
Asparagine	7.745E-05	3.984E-05	8.506E-05	3.484E-05	0.536
Aspartic acid	4.126E-04	1.326E-04	4.189E-04	8.649E-05	0.750
Serine	7.230E-04	1.622E-04	7.298E-04	1.319E-04	0.808
Alanine	1.947E-02	6.607E-03	2.286E-02	4.579E-03	0.116
4-Hydroxyproline	3.433E-04	1.358E-04	4.301E-04	1.588E-04	0.206
Glycine	4.413E-04	9.391E-05	4.494E-04	8.944E-05	0.750
Citicoline	7.903E-06	1.404E-05	1.063E-05	1.979E-05	0.911
Glutamine	2.953E-02	3.410E-03	3.088E-02	4.163E-03	0.377
Threonine	3.439E-03	7.568E-04	3.491E-03	8.851E-04	0.837
Dimethylglycine	4.708E-03	1.097E-03	4.651E-03	8.881E-04	0.750
Methionine sulfoxide	1.046E-04	4.747E-05	9.511E-05	3.397E-05	0.319
Glutamic acid	3.490E-03	1.746E-03	3.623E-03	1.366E-03	0.694
Citrulline	7.294E-03	1.317E-03	6.693E-03	8.798E-04	0.135
Guanosine	3.967E-06	1.484E-05	2.479E-07	1.052E-06	0.851
monophosphate					
Proline	2.485E-01	4.956E-02	2.753E-01	8.652E-02	0.442
Ornithine	4.251E-03	1.102E-03	4.110E-03	9.098E-04	0.750
2-Aminobutyric acid	1.918E-02	5.096E-03	2.011E-02	7.018E-03	0.955
Lysine	3.186E-02	3.671E-03	3.258E-02	4.485E-03	0.694
Histidine	9.708E-03	1.656E-03	1.042E-02	1.969E-03	0.420
Adenosine	4.911E-04	2.127E-04	6.759E-04	3.769E-04	0.206
monophosphate					
Uracil	3.357E-04	1.014E-04	3.380E-04	1.211E-04	0.925
Argininosuccinic acid	4.759E-06	1.781E-05	1.811E-06	5.311E-06	0.792
Thymidine	3.525E-04	3.950E-05	3.977E-04	7.492E-05	0.125
monophosphate					
Arginine	5.495E-02	1.148E-02	5.614E-02	9.900E-03	0.512
Creatine	3.491E-02	1.206E-02	3.948E-02	1.639E-02	0.587
Cytosine	2.301E-05	3.634E-05	1.278E-05	1.807E-05	0.606
Hypoxanthine	6.302E-04	1.968E-04	5.695E-04	2.767E-04	0.357
Uridine	5.871E-03	1.262E-03	6.104E-03	1.281E-03	0.377
Niacinamide	2.860E-04	1.064E-04	3.877E-04	1.284E-04	< 0.05
Adenosine 3',5'-cyclic	1.437E-05	2.063E-05	1.015E-05	2.065E-05	0.638
monophosphate					
Guanosine	0	NA	6.817E-06	1.730E-05	0.165
Inosine	0	NA	2.646E-05	4.495E-05	< 0.05
Pantothenic acid	1.674E-04	8.621E-05	1.582E-04	7.059E-05	0.955
Adenine	4.656E-05	3.952E-05	4.169E-05	2.024E-05	0.849
Tyrosine	8.111E-02	1.878E-02	8.979E-02	1.276E-02	0.235
Adenosine	8.633E-06	1.771E-05	1.784E-05	3.288E-05	0.582
Epinephrine	9.644E-05	3.229E-05	9.507E-05	2.436E-05	0.985
Asymmetric	2.457E-06	9.195E-06	0	NA	0.415
dimethylarginine					
Phenylalanine	7.267E-01	9.136E-02	7.492E-01	7.121E-02	0.267
Kynurenine	4.298E-03	8.353E-04	4.559E-03	9.591E-04	0.808
Acetyl-L-carnitine	1.013E-01	1.947E-02	8.699E-02	1.874E-02	< 0.05
Tryptophan	2.859E-01	5.521E-02	2.990E-01	4.335E-02	0.357
2-Ketoglutaric acid	3.563E-04	1.234E-04	3.979E-04	8.680E-05	0.091
Malic acid	5.997E-04	1.276E-04	6.457E-04	1.697E-04	0.640
Isocitric acid	3.879E-03	8.329E-04	4.398E-03	7.126E-04	0.067
Pyruvic acid	1.951E-04	1.178E-04	2.225E-04	1.234E-04	0.362
Lactic acid	1.959E-02	5.195E-03	1.832E-02	4.893E-03	0.442
Uric acid	1.208E-02	1.914E-03	1.146E-02	2.774E-03	0.536
Citric acid	4.857E-02	5.312E-03	5.251E-02	5.954E-03	0.107
Succinic acid	2.037E-04	1.572E-04	1.987E-04	1.424E-04	1.000
Xanthine	1.058E-05	2.699E-05	7.473E-05	2.926E-04	0.675

P-value is based on Mann-Whitney U-test. Significant is at the 5% level. Peak areas of individual metabolites were normalized against the peak area of the internal standards, and the resulting values were represented as relative areas.

ALT, alanine aminotransferase; MS, metabolic syndrome; NA, not applicable; NAFLD, nonalcoholic fatty liver disease, NS, not significant; SD, standard deviation.

### Correlations between clinical parameters and significant metabolites for NAFLD having elevation of ALT

Spearman rank coefficients for clinical parameters and metabolites with statistically significant differences between subjects with and without NAFLD having elevation of ALT in MS are shown in Table 6. Niacinamide levels correlated significantly with ALT, NAFIC score, and NAFLD having elevation of ALT (*p* <0.05 for all). Inosine levels correlated significantly with ALT, HOMA-IR, and NAFLD having elevation of ALT (*p* < 0.05, *p* <0.01, and *p* <0.05, respectively). Acetyl-L-carnitine levels correlated significantly with IGT and NAFLD having elevation of ALT (*p* < 0.05 for both).

**Table 6:** Spearman rank coefficients for clinical parameters and metabolites those showed a statistically significant difference between subjects with and without NAFLD having elevation of ALT in MS.

	Niacinamide	Inosine	Acetylcarnitine
BMI	-0.001	0.181	-0.016
WC	-0.082	0.182	-0.027
Hypertension	-0.021	0.180	0.113
Dyslipidemia	-0.091	-0.227	0.189
IGT	-0.132	-0.173	0.445*
ALT	0.368*	0.374*	-0.252
AST	0.302	0.253	-0.259
GGT	0.151	-0.276	-0.079
UA	-0.133	-0.018	0.101
HOMA-IR	0.269	0.472**	-0.222
NAFIC score	0.350*	0.189	-0.207
FIB-4 index	0.120	0.060	-0.310
Fatty liver	0.113	0.229	0.113
NAFLD with elevation of ALT	0.368*	0.421*	-0.355*

*p < 0.05

**p < 0.01.

ALT, alanine aminotransferase; AST, aspartate aminotransferase; BMI, body mass index; GGT, gamma-glutamyl transpeptidase; HOMA-IR: homeostasis model assessment of insulin resistance; IGT, impaired glucose tolerance; MS, metabolic syndrome; NAFLD, nonalcoholic fatty liver disease; UA, uric acid; WC, waist circumference.

### Comparison of sulfur metabolites between subjects with and without NAFLD having elevation of ALT in 32 subjects with MS

A comparison of 17 sulfur metabolites that was associated sulfur metabolic pathways between subjects with and without NAFLD having elevation of ALT in 32 subjects with MS are shown in Table 7. There were no differences in sulfur metabolites between subjects with and without NAFLD having elevation of ALT.

**Table 7:** Comparison of 17 sulfur metabolites between subjects with and without NAFLD having elevation of ALT in 32 subjects with MS.

Compound name	Relative area				
	Subjects without NAFLD having elevation of ALT (n = 14)		Subjects with NAFLD having elevation of ALT (n = 18)		*p*-value
	Mean	SD	Mean	SD	
Glucose	2.52E-03	4.43E-04	2.49E-03	4.61E-04	0.896
Methionine	7.67E-04	2.27E-04	8.10E-04	3.58E-04	0.640
Serine	0	NA	2.83E-04	1.20E-03	0.526
Histidine	6.06E-03	9.06E-04	5.94E-03	1.16E-03	0.694
Cys-bimane	1.37E-03	1.80E-04	1.51E-03	4.48E-04	0.613
Cys-S-bimane	1.18E-03	3.86E-04	1.06E-03	4.41E-04	0.283
GS-bimane	7.00E-05	5.69E-05	6.70E-05	5.60E-05	0.675
GS-S-bimane	3.47E-06	1.30E-05	1.79E-06	7.60E-06	0.851
Sulfite-bimane	1.69E-04	2.18E-04	1.26E-04	5.67E-05	0.955
SDB	8.70E-03	5.58E-03	8.67E-03	3.77E-03	0.319
Thiosulfate-bimane	2.59E-02	1.43E-02	2.52E-02	1.36E-02	0.955
Ergothioneine-bimane	2.30E-02	9.88E-03	2.43E-02	1.11E-02	0.750
Cystathionine	3.88E-04	8.51E-05	3.99E-04	8.46E-05	0.925
GSSG [M+2H]2+	3.10E-05	3.20E-05	2.85E-05	3.33E-05	0.877
Ergothioneine	3.68E-05	1.38E-04	0	NA	0.415
S-sulfocysteine	1.98E-06	5.23E-06	0	NA	0.177
Lactic acid	1.17E-02	3.02E-03	1.17E-02	3.10E-03	0.894

*P*-value is based on Mann-Whitney U-test. Significant is at the 5% level. Peak areas of individual metabolites were normalized against the peak area of the internal standards, and the resulting values were represented as relative areas.

ALT, alanine aminotransferase; Cys, cysteine; GS, glutathione; GSSG [M+2H]2+, glutathione oxide sulfide; MS, metabolic syndrome; NA, not applicable; NAFLD, nonalcoholic fatty liver disease; NS, not significant; S, sulfide; SD, standard deviation; SDB, sulfide dibimane.

## Discussion

The aim of the present study was to clarify the differences in clinical factors associated with NAFLD having elevation of ALT among subjects with Non-MS, Pre-MS, and MS, and to measure differences in metabolites between MS subjects with and without NAFLD having elevation of ALT. The present study showed that the prevalence of NAFLD having elevation of ALT was progressively greater in subjects with Non-MS, Pre-MS, and MS, however, independent predictors for NAFLD having elevation of ALT differed among subjects with MS, Pre-MS, and Non-MS. Additionally, there were significant differences in levels of several metabolites between subjects with and without NAFLD having elevation of ALT in spite of the subjects who belong to same MS. To our knowledge, ours is the first study to clarify the differences in subjects with NAFLD having elevation of ALT at various stage of MS, as well as measuring differences in metabolites with respect to NAFLD having elevation of ALT in the context of medical check-ups.

We demonstrated that values of physical measurements such as BMI and WC, and almost factors related to hypertension, dyslipidemia, and IGT were progressively greater in the Non-MS, Pre-MS, MS groups, in accordance with previous reports that BMI, WC, blood pressure, HOMA-IR, and others increased with the number of MS components [17–19]. Liver enzymes such as ALT and AST, and the prevalence of NAFLD and NAFLD having elevation of ALT increased with progression from Non-MS to Pre-MS to MS. These results suggest that the onset of NAFLD and elevation of liver enzyme were strongly associated with MS components such as obesity, including visceral fat and lifestyle-related diseases. Furthermore, the prevalence of NAFLD and NAFLD having elevation of ALT among subjects except the MS group were 25.3% and 8.4%, respectively. This prevalence was not low, and we cannot ignore this fact.

We showed that independent predictors of NAFLD having elevation of ALT varied among Non-MS, Pre-MS, and MS groups. The prevalence of dyslipidemia and IGT in the Non-MS group was lower than that of the MS group; however, LDL, HbA1c, and UA were significant factors of NAFLD having elevation of ALT in the Non-MS group. These results suggest that, even in non-obese individuals, paying attention to dyslipidemia and IGT is necessary. Hyperuricemia is associated with an elevated risk of developing impaired fasting glucose [20]. IGT due to insulin resistance may lead to hyperinsulinemia, which increases uric acid concentrations by reducing renal uric acid secretion and accumulating substrates for uric acid production [21, 22]. Hyperuricemia was reported to be associated with histological liver damage in patients with NAFLD [23]. These findings suggest that our study might indicate that not only HbA1c but also UA are significant risk factors for NAFLD having elevation of ALT in the Non-MS group.

In the MS group, IGT was a significant independent predictor of an increase prevalence of NAFLD having elevation of ALT in accordance with previous reports that DM and insulin resistance are associated with NAFLD [24, 25]. Obesity and visceral adipose tissue are risk factors for insulin resistance. In particular, excessive visceral fat accumulation releases various bioactive substances known as inflammatory adipokines, include interleukin-6, tumor necrosis factor-α, macrophage chemoattractant protein-1, and resistin [26, 27]. Therefore, visceral fat accumulation is thought to play an important role in the development of NAFLD [28–33]. These findings support our result that the prevalence of NAFLD and NAFLD having elevation of ALT in MS group were higher than in other groups.

Conversely, HDL-C was a significant independent predictor of decreased risk for NAFLD having elevation of ALT in the Non-MS and MS groups. Levels of TG, IGT, and HDL-C were significantly different between subjects with and without NAFLD having elevation of ALT in the Non-MS and MS groups. NAFLD is strongly associated with dyslipidemia, including decreased HDL-C levels and increased TG levels [34, 35]. TG is synthesized from free fatty acids. Excess energy, decreased lipolysis in adipose tissue, increased lipogenesis in the liver, and insulin resistance that suppresses lipolysis and increases de novo lipogenesis may induce increased free fatty acid levels [36–38]. The main component of very low-density lipoprotein (VLDL) in liver is TG, and increased VLDL and IGT in diabetes causes decreased HDL-C levels [39]. Therefore, our study might show that HDL is a significant independent predictor of decreased prevalence of NAFLD having elevation of ALT.

In the Pre-MS group, hypertension was a significant independent predictor of decreased prevalence of NAFLD having elevation of ALT. However, there was no significant difference in the prevalence of NAFLD having elevation of ALT between subjects without hypertension and subjects having hypertension without medication. In recent reports, angiotensin II receptor blockers may suppress liver fibrosis with NAFLD including non-alcoholic steatohepatitis [40–42]. Our results might be affected by use of antihypertensive agents. Further studies are needed to elucidate the association between NAFLD and antihypertensive agents because we did not investigate the type of antihypertensive agents.

Metabolomics involves the measurement of large numbers of low-molecular-weight metabolites, including sugars, amino acids, and hormones. Although several studies have provided insight into the pathogenesis of NAFLD [43–46], to date, no specific biomarker that could identify NAFLD has been found using metabolomics. We found that the levels of metabolites such as inosine and nicotinamide were significantly higher in subjects with NAFLD having elevation of ALT than those in subjects without NAFLD having elevation of ALT. Inosine is found in meat and is an organic compound with a nucleoside-like structure. Consuming large amounts of food containing inosine may increase uric acid levels because inosine is metabolized to uric acid. The elevation of uric acid levels is associated with NAFLD in several cohort studies [47, 48]. Nicotinamide is a B-complex vitamin that participates in energy production and metabolism of sugar, lipids, and proteins as a coenzyme of dehydrogenase. High dietary intake of nicotinamide is unfavorable because the accumulation of nicotinamide may cause liver dysfunction [49]. This suggests that elevated serum levels of inosine and nicotinamide may be associated with NAFLD. Conversely, acetyl-L-carnitine levels were significantly lower in subjects with NAFLD having elevation of ALT than those in subjects without NAFLD having elevation of ALT in our study. Acetyl-L-carnitine is an acetylated derivative of L-carnitine that participates in oxidative metabolism. Decreased L-carnitine levels were associated with insulin resistance and L-carnitine supplementation in rats and mice improved metabolic function and NAFLD [50, 51]. Our findings suggest that lower levels of acetyl-L-carnitine may be associated with NAFLD. Recently, reactive sulfur species (RSS) have been recognized to be endogenously produced in abundance in many species [52, 53]. They occur in diverse polysulfide forms with unique redox-active or reactive chemical properties [54–56]. Sulfur metabolomics was developed to investigate the sulfur metabolic pathways associated with RSS [52, 53, 57]. Although there was no sulfur metabolite that showed statistically significantly different levels between subjects with and without NAFLD having elevation of ALT in our study, the additional results showed that there was a tendency of difference in levels of S-sulfocysteine between subjects having NAFLD with normal ALT levels and those having NAFLD with elevation of ALT levels (*p* = 0.072, S1 Table). Although the influence of S-sulfocysteine on the liver is unclear, there is a possibility that sulfites and its derivatives induce oxidative stress [58, 59] and disturb mitochondrial function in rat experiments [60, 61]. Metabolomics in our study identified three metabolites that were significantly correlated with NAFLD having elevation of ALT, and one sulfur metabolite had the possibility of differentiating between subjects having NAFLD with elevation of ALT levels and those having NAFLD with normal ALT levels. These results suggest that metabolomics may become a useful screening test for NAFLD in individuals with MS during medical check-ups in the future.

Although the HOMA-IR and NAFIC scores effectively discriminated between subjects with and without NAFLD having elevation of ALT in our study, they were within the range of normal in almost all subjects. Therefore, not only persons whose HOMA-IR or NAFIC scores are high but also those whose HOMA-IR or NAFIC scores are within standard values should be carefully assessed during medical check-ups.

The present study had several limitations. First, it was a single-center study, and therefore may be subject to selection bias. For this reason, we instituted strict inclusion and exclusion criteria. Multi-center studies are needed to validate our findings. Second, different results may be found in patients who go to the hospital for NAFLD and those who are found to have NAFLD during medical check-ups because most of the participants in the present study were healthy individuals without symptoms. Further investigations of the differences between these groups are required. Third, we did not obtain information regarding treatments for hypertension and DM, diets (e.g., volume and contents including vegetable and fruits), and total caloric intake. Finally, the number of subjects who were investigated regarding metabolomics was small because metabolomics is not usually included in medical check-ups. Further studies are necessary to resolve these limitations.

In conclusion, we demonstrated that the prevalence of NAFLD having elevation of ALT was progressively higher in Non-MS, Pre-MS and MS groups. Significant independent predictors for NAFLD having elevation of ALT were different among the three groups. Not only liver scoring systems such as the HOMA-IR and NAFIC score but also several metabolites may help identify the risk of NAFLD in individuals with MS.

## Supporting information

S1 Checklist (DOC)

S1 Table Comparison of 17 sulfur metabolites between subjects with NAFLD having elevation of ALT and subjects with NAFLD having standard values of ALT in 26 subjects with MS. (DOCX)
