# Supplementary material for: Differences among patients with and without nonalcoholic fatty liver disease having elevated alanine aminotransferase levels at various stages of metabolic syndrome
Source: PLoS One. 2020 Aug 31;15(8):e0238388. doi: 10.1371/journal.pone.0238388 (PMC7458345; doi:10.1371/journal.pone.0238388)
Supplement: S1 Checklist — (DOC) [file pone.0238388.s001.doc]

STROBE Statement—Checklist of items that should be included in reports of ***cross-sectional studies***

|  | Item No | Recommendation |
| --- | --- | --- |
| **Title and abstract** | 1 | (*a*) Indicate the study’s design with a commonly used term in the title or the abstract  Page1 |
|  |  | (*b*) Provide in the abstract an informative and balanced summary of what was done and what was found  Page2-4 |
| Introduction | | |
| Background/rationale | 2 | Explain the scientific background and rationale for the investigation being reported  Page4-5 |
| Objectives | 3 | State specific objectives, including any prespecified hypotheses  Page5 |
| Methods | | |
| Study design | 4 | Present key elements of study design early in the paper  Page5 |
| Setting | 5 | Describe the setting, locations, and relevant dates, including periods of recruitment, exposure, follow-up, and data collection  Page5 |
| Participants | 6 | (*a*) Give the eligibility criteria, and the sources and methods of selection of participants  Page5-6 |
| Variables | 7 | Clearly define all outcomes, exposures, predictors, potential confounders, and effect modifiers. Give diagnostic criteria, if applicable  Page5-8 |
| Data sources/ measurement | 8* | For each variable of interest, give sources of data and details of methods of assessment (measurement). Describe comparability of assessment methods if there is more than one group  Page6-10 |
| Bias | 9 | Describe any efforts to address potential sources of bias  Page30-31 |
| Study size | 10 | Explain how the study size was arrived at  Page5 |
| Quantitative variables | 11 | Explain how quantitative variables were handled in the analyses. If applicable, describe which groupings were chosen and why  Page6,8-9 |
| Statistical methods | 12 | (*a*) Describe all statistical methods, including those used to control for confounding  Page9-10 |
|  |  | (*b*) Describe any methods used to examine subgroups and interactions  Page6-7,9-10 |
|  |  | (*c*) Explain how missing data were addressed  NA |
|  |  | (*d*) If applicable, describe analytical methods taking account of sampling strategy  Page9-10 |
|  |  | (*e*) Describe any sensitivity analyses  NA |
| Results | | |
| Participants | 13* | (a) Report numbers of individuals at each stage of study—eg numbers potentially eligible, examined for eligibility, confirmed eligible, included in the study, completing follow-up, and analysed  Page10-13 |
|  |  | (b) Give reasons for non-participation at each stage  Page5-7,9 |
|  |  | (c) Consider use of a flow diagram  Page5, Fig1 |
| Descriptive data | 14* | (a) Give characteristics of study participants (eg demographic, clinical, social) and information on exposures and potential confounders  Page10-14 |
|  |  | (b) Indicate number of participants with missing data for each variable of interest  NA |
| Outcome data | 15* | Report numbers of outcome events or summary measures  Page14-22 |
| Main results | 16 | (*a*) Give unadjusted estimates and, if applicable, confounder-adjusted estimates and their precision (eg, 95% confidence interval). Make clear which confounders were adjusted for and why they were included  Page14-17 |
|  |  | (*b*) Report category boundaries when continuous variables were categorized  Page14-17 |
|  |  | (*c*) If relevant, consider translating estimates of relative risk into absolute risk for a meaningful time period  Page14-17 |
| Other analyses | 17 | Report other analyses done—eg analyses of subgroups and interactions, and sensitivity analyses  Page17-23 |
| Discussion | | |
| Key results | 18 | Summarise key results with reference to study objectives  Page24-25,31 |
| Limitations | 19 | Discuss limitations of the study, taking into account sources of potential bias or imprecision. Discuss both direction and magnitude of any potential bias  Page30-31 |
| Interpretation | 20 | Give a cautious overall interpretation of results considering objectives, limitations, multiplicity of analyses, results from similar studies, and other relevant evidence  Page25-30 |
| Generalisability | 21 | Discuss the generalisability (external validity) of the study results  Page31 |
| Other information | | |
| Funding | 22 | Give the source of funding and the role of the funders for the present study and, if applicable, for the original study on which the present article is based  NA |

*Give information separately for exposed and unexposed groups.

**Note:** An Explanation and Elaboration article discusses each checklist item and gives methodological background and published examples of transparent reporting. The STROBE checklist is best used in conjunction with this article (freely available on the Web sites of PLoS Medicine at http://www.plosmedicine.org/, Annals of Internal Medicine at http://www.annals.org/, and Epidemiology at http://www.epidem.com/). Information on the STROBE Initiative is available at www.strobe-statement.org.
