# Supplementary material for: Differences among patients with and without nonalcoholic fatty liver disease having elevated alanine aminotransferase levels at various stages of metabolic syndrome
Source: PLoS One. 2020 Aug 31;15(8):e0238388. doi: 10.1371/journal.pone.0238388 (PMC7458345; doi:10.1371/journal.pone.0238388)
Supplement: S1 Table — (DOCX) [file pone.0238388.s002.docx]

**S1 Table** Comparison of 17 sulfur metabolites between subjects with NAFLD having elevation of ALT and subjects with NAFLD having standard values of ALT in 26 subjects with MS.

| Compound name | Relative area | |  |  | |  | |  |
| --- | --- | --- | --- | --- | --- | --- | --- | --- |
|  | Subjects with  NAFLD having  elevation of ALT  (n = 18) | | | | Subjects with  NAFLD having  standard values of ALT  (n = 8) | | | *p*-value |
|  | Mean | SD | | | Mean | | SD |  |
| Glucose | 2.49E-03 | 4.61E-03 | | | 2.42E-03 | | 4.76E-04 | 0.807 |
| Methionine | 8.10E-04 | 3.58E-04 | | | 7.29E-04 | | 2.21E-04 | 0.531 |
| Serine | 2.83E-04 | 1.20E-03 | | | 0 | | NA | 0.631 |
| Histidine | 5.94E-03 | 1.16E-03 | | | 6.27E-03 | | 9.57E-04 | 0.397 |
| Cys-bimane | 1.51E-03 | 4.48E-04 | | | 1.38E-03 | | 1.93E-04 | 0.765 |
| Cys-S-bimane | 1.06E-03 | 4.41E-04 | | | 1.34E-03 | | 4.02E-04 | 0.148 |
| GS-bimane | 6.70E-05 | 5.60E-05 | | | 6.93E-05 | | 5.46E-05 | 0.697 |
| GS-S-bimane | 1.79E-06 | 7.60E-06 | | | 6.08E-06 | | 1.72E-05 | 0.582 |
| Sulfite-bimane | 1.26E-04 | 5.67E-05 | | | 1.24E-04 | | 4.06E-05 | 0.765 |
| SDB | 8.67E-03 | 3.76E-03 | | | 1.02E-02 | | 7.08E-03 | 0.892 |
| Thiosulfate-biman | 2.52E-02 | 1.36E-02 | | | 3.24E-02 | | 1.44E-02 | 0.196 |
| Ergothioneine-bimane | 2.43E-02 | 1.11E-02 | | | 2.13E-02 | | 9.11E-03 | 0.567 |
| Cystathionine | 3.99E-04 | 8.46E-05 | | | 3.97E-04 | | 9.75E-05 | 0.892 |
| GSSG [M+2H]2+ | 2.85E-05 | 3.33E-05 | | | 1.98E-05 | | 1.84E-05 | 0.567 |
| Ergothioneine | 0 | NA | | | 6.44E-05 | | 1.82E-04 | 0.279 |
| S-sulfocysteine | 0 | NA | | | 3.46E-06 | | 6.71E-06 | 0.071 |
| Lactic acid | 1.17E-02 | 3.10E-03 | | | 1.30E-02 | | 3.14E-03 | 0.216 |

*P*-value is based on Mann-Whitney U-test. Significant is at the 5% level. Peak areas of individual metabolites were normalized against the peak area of the internal standards, and the resulting values were represented as relative areas.

ALT, alanine aminotransferase; Cys, cysteine; GS, glutathione; GSSG [M+2H]2+, glutathione oxide sulfide; NA, not applicable; NAFLD, nonalcoholic fatty liver disease; NS, not significant; S, sulfide; SD, standard deviation; SDB, sulfide dibimane.
